# N‐Truncated Superoxide Dismutase‐1 in Cerebrospinal Fluid Is Folded and Active

**DOI:** 10.1111/jnc.70382

**Published:** 2026-02-10

**Authors:** Laura Leykam, Karin M. E. Forsberg, Peter M. Andersen, Thomas Brännström, Sophia Weiner, John Rönnholm, Kaj Blennow, Henrik Zetterberg, Stefan L. Marklund, Johan Gobom, Per Zetterström

**Affiliations:** ^1^ Department of Medical Biosciences, Clinical Chemistry Umeå University Umeå Sweden; ^2^ Department of Clinical Sciences, Neurosciences Umeå University Umeå Sweden; ^3^ Department of Medical Biosciences, Pathology Umeå University Umeå Sweden; ^4^ Institute of Neuroscience and Physiology, Department of Psychiatry and Neurochemistry University of Gothenburg Mölndal Sweden; ^5^ Clinical Neurochemistry Lab, Institute of Neuroscience and Physiology Sahlgrenska University Hospital Mölndal Sweden; ^6^ Paris Brain Institute, ICM, Pitié‐Salpêtrière Hospital, Sorbonne University Paris France; ^7^ Neurodegenerative Disorder Research Center, Division of Life Sciences and Medicine, and Department of Neurology, Institute on Aging and Brain Disorders University of Science and Technology of China and First Affiliated Hospital of USTC Hefei People's Republic of China; ^8^ Department of Neurodegenerative Disease UCL Institute of Neurology London UK; ^9^ UK Dementia Research Institute, UCL London UK; ^10^ Hong Kong Center for Neurodegenerative Diseases, InnoHK Hong Kong China; ^11^ Wisconsin Alzheimer's Disease Research Center University of Wisconsin School of Medicine and Public Health, University of Wisconsin‐Madison Madison Wisconsin USA; ^12^ Department of Pathology and Laboratory Medicine University of Wisconsin School of Medicine and Public Health Madison Wisconsin USA; ^13^ Centre for Brain Research Indian Institute of Science Bangalore India

**Keywords:** amyotrophic lateral sclerosis, cerebrospinal fluid, posttranslational modification, superoxide dismutase‐1

## Abstract

Mutations in the antioxidant enzyme superoxide dismutase‐1 (SOD1) are a well‐established cause of amyotrophic lateral sclerosis (ALS). The mutations promote SOD1 misfolding, resulting in protein aggregation and motor neuron degeneration. SOD1 is normally a structurally stable enzyme, and the mechanisms underlying SOD1 misfolding remain poorly understood. Approximately one third of SOD1 in cerebrospinal fluid (CSF) exhibits an N‐terminal truncation, the biological significance of which remains unclear. This is remarkable given the dramatic effects ALS‐linked C‐terminal truncations have on the enzyme. In this study, we identified the truncation site and investigated its impact on SOD1 stability and enzymatic activity. Edman degradation revealed the cleavage site between Asn‐26 and Gly‐27, generating a 26‐residue peptide that was confirmed by mass spectrometry. We analyzed postmortem tissues from different parts of the central nervous system (CNS), including the choroid plexus, and found only trace amounts of N‐terminally truncated SOD1. Biochemical characterization of the SOD1 in CSF was done by size exclusion chromatography, ion exchange chromatography, and mass spectrometry. Our findings demonstrate that SOD1 in CSF retains full enzymatic activity, that the N‐terminally truncated variant is mainly present in heterodimers with native SOD1 subunits, and that the dimer remains folded and active, with both fragments of the truncated SOD1 fixed after proteolysis. Truncated SOD1 was absent in human plasma. In mice, only transgenically expressed human SOD1 underwent truncation in CSF, whereas endogenous murine SOD1 remained intact. Lastly, the N‐terminal truncation does not induce misfolding, unlike the destabilizing effects observed with C‐terminal truncations. The location where the truncation takes place and the underlying mechanism could not be identified. Whether the N‐truncated SOD1 variant contributes to ALS pathogenesis remains to be determined.

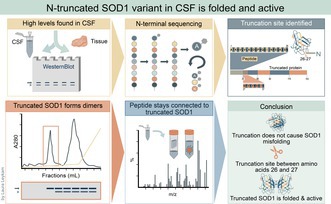

## Graphical abstract

Superoxide dismutase 1 (SOD1) mutations cause amyotrophic lateral sclerosis (ALS) through mechanisms involving protein misfolding and aggregation. While C‐terminal truncations of SOD1 promote neurotoxicity, our study reveals that approximately one‐third of SOD1 in cerebrospinal fluid (CSF) is N‐terminally truncated—a striking observation given the structural importance of this region. The cleavage site was between Asn‐26 and Gly‐27. Both fragments stay fixed within the folded β‐barrel architecture and retain full enzymatic activity. Importantly, truncated SOD1 was abundant in CSF but nearly absent in CNS tissue, and its presence was independent of ALS status. We found no correlation between N‐terminal truncation and misfolded SOD1 levels, suggesting no contribution to ALS.

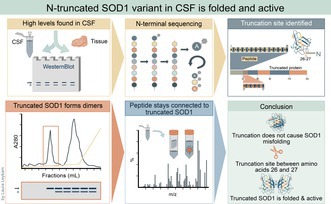

**Abbreviations**
ALS: amyotrophic lateral sclerosis
AU: arbitrary units
BSA: bovine serum albumin
CNS: central nervous system
CSF: cerebrospinal fluid
EDTA: ethylenediaminetetraacetic acid
ELISA: enzyme‐linked immunosorbent assay
FA: formic acid
FDR: false discovery rate
IAM: iodoacetamide
PBS: phosphate‐buffered saline
RRID: research resource identifier
RT: room temperature
SD: standard deviation
SEC: size exclusion chromatography
SOD1: superoxide dismutase1

## 1 Introduction

Superoxide dismutase‐1 (SOD1, EC 1.15.1.1) is a ubiquitously expressed enzyme that catalyzes the conversion of superoxide radicals into hydrogen peroxide and molecular oxygen in human cells (Marklund 1984; McCord and Fridovich 1969). SOD1 is predominantly cytosolic, but also localizes to the nucleus and mitochondrial intermembrane space and makes up around 0.16% of the total protein content in the human central nervous system (CNS) (Forsberg et al. 2010; Leykam et al. 2025). Functionally, SOD1 forms a 32 kDa homodimer, with each subunit comprising 153 highly conserved amino acids, each subunit containing a copper ion and a zinc ion. The copper ion is alternatively oxidized and reduced as part of the dismutation reaction while the zinc ion mediates the correct folding of the SOD1 monomer: a conformation of an eight‐stranded Greek key β‐barrel, which are connected via seven loops (Parge et al. 1992). Its exceptional structural stability (Stathopulos et al. 2006; Weser et al. 1989) is maintained by the intramolecular disulfide bond between cysteine residues 57 and 146 in each subunit, the dimer formation, and the coordination of primarily the zinc ion but also the copper ion to histidine and aspartic acid residues in the electrostatic loop and β‐strands IV, V and VII (Eleutherio et al. 2021).

In 1993, mutations in the SOD1 gene were identified as the first genetic cause of amyotrophic lateral sclerosis (ALS) (Rosen et al. 1993). Since then, over 230 mutations in *SOD1* have been reported in ALS patients worldwide, usually as a dominant trait with complete or reduced penetrance, but recessive inheritance and *de novo* mutations have also been found (Andersen et al. 1995; Müller et al. 2022). Accumulated evidence indicates that *SOD1* mutations destabilize the protein, which leads to unfolding and misfolding of the SOD1 monomers. These misfolded species are prone to aggregation, and SOD1 inclusions in motor neurons are considered a hallmark of SOD1‐linked ALS (Kato et al. 2000). Two different tertiary structures of SOD1 aggregates referred to as strains A and B have been identified in transgenic mice overexpressing human SOD1 (Bergh et al. 2015). Indeed, the pathogenic role of such SOD1 aggregates has been investigated by spinal cord inoculation experiments in transgenic ALS model mice and suggest that the human SOD1 aggregates fulfill characteristics of ALS‐transmitting prions (Ayers et al. 2014; Bidhendi et al. 2016). Our previous work demonstrated that aggregates isolated from spinal ventral horns from an ALS patient carrying the G127X mutation likewise transmit SOD1 aggregation and premature ALS‐like disease (Ekhtiari Bidhendi et al. 2018). However, SOD1 inclusions have also been reported in ALS cases lacking *SOD1* mutations (Bosco et al. 2010; Forsberg et al. 2011, 2019, 2010; Grad et al. 2011, 2014), which could imply a greater role for SOD1 in ALS pathogenesis.

The majority of ALS‐associated *SOD1* mutations are missense variants but over 30 nonsense mutations have been reported which result in insertions and/or deletions of nucleotides with a premature stop codon and a predicted shortened C‐terminally truncated mutant protein (ALSoD, (Wroe et al. 2008)). The best studied of these nonsense mutations is the G127X mutation with an insertion of four base pairs in codon 127 causing a frameshift and the introduction of five new amino acids and a premature stop codon in position 133 (Andersen et al. 1997). The G127X protein is severely destabilized, and most is rapidly degraded in vivo (Jonsson et al. 2004), resulting in low SOD1 activity in erythrocytes of approximately 40% compared to control individuals. This suggests that the mutation has a dominant negative effect on the enzymatic activity of SOD1 (Andersen et al. 1997; Jonsson et al. 2004). In transgenic mice expressing the G127X protein, large amounts of aggregated protein accumulate in terminal stages and aggregated G127X protein is also detected in the CNS in human patients at autopsy (Jonsson et al. 2004).

Previously, we identified a low‐molecular‐weight SOD1 variant in human cerebrospinal fluid (CSF) samples in addition to the native full‐length human SOD1 protein on western blots (Jacobsson et al. 2001). We concluded that it was an N‐truncated form of SOD1 based on antibodies covering the sequence of the SOD1 molecule and found that the truncated form represented ~30% of all SOD1 in the CSF. Data from Gertsman et al. (2019) show that a peptide from the N‐terminal end of SOD1 is elevated in CSF from ALS patients. This supports our conclusion that SOD1 is truncated in the N‐terminus. The N‐truncated form was detected in ALS patients with and without SOD1 mutations as well as in controls and was hence not caused by any mutation. Further, analysis of SOD1 protein and SOD1 enzymatic activity suggested that the truncated SOD1 was fully enzymatically active. Considering the pronounced neurotoxicity of C‐terminally truncated SOD1 variants such as G127X, the presence of a large fraction of N‐truncated SOD1 in CSF is remarkable.

In this study, we further characterize the N‐terminally truncated SOD1 variant. High levels were found in CSF whereas only traces of truncated SOD1 were found in the spinal cord, brain, liver, kidney, gastrocnemius muscle, and blood plasma. We identified the cleavage site and showed that the N‐terminal peptide fragment stays in situ, attached by noncovalent binding to the truncated subunit. The SOD1 dimer remains natively folded, thereby retaining enzymatic activity.

## 2 Material and Methods

### 2.1 Patients, Control Participants, and Collection of CSF

The participating patients and the protocol for CSF collection have been previously described (Leykam et al. 2024). Briefly, with consent from the patient and the Research Ethics Committee at Umeå University (FEK 1994 dnr 94–135 with later amendments) adhering to the Declaration of Helsinki (WMA 1964), patients seen at the Department of Neurology, Umeå University Hospital were asked to donate to medical research any surplus CSF remaining after the diagnostic clinical tests had been performed. CSF was collected via gravity flow into polypropylene tubes, aliquoted into 1.5 mL tubes that were then immediately frozen to −80°C without prior centrifugation. The ALS patients were diagnosed according to the European Federation of Neurological Societies diagnostic algorithm for managing ALS (Andersen et al. 2012). Mutation analysis of *SOD1* and several other ALS‐causing genes was performed as described (Müller et al. 2022). CSF samples were obtained between 1995 and 2023. The control subjects were patients with a variety of nonmotor neuron disease diagnoses seen at the neurological department at Umeå University Hospital; the most common diagnoses were headache, paresthesia, and polyneuropathy. Some of the procedures described herein required larger volumes of CSF not possible to collect from a single individual. For these experiments, CSF was pooled from several subjects, and the pooled CSF was stored at −80°C between experiments.

### 2.2 Autopsy Tissues

Following informed consent from patients or next of kin, tissue specimens from ALS patients and controls were saved and immediately frozen at −80°C (Forsberg et al. 2011, 2010). The protocol was approved by the Research Ethics Committee at Umeå University (dnr 2014–17‐31 M) as well as the Umeå Regional Ethical Review Board and adhered to the principles of the Declaration of Helsinki.

### 2.3 Blood Plasma

Venous blood was drawn via spontaneous flow through 18G needles to minimize hemolysis from five healthy anonymous donors. The blood was mixed with K_2_‐ethylenediaminetetraacetic acid (EDTA), the tubes were inverted over 10 times to mix the blood and anticoagulant, and then centrifuged at 2000 × *g* for 10 min at room temperature (RT, 22°C) to collect the plasma and separated erythrocytes. Absorbance was measured at 415 nm on a CLARIOstar plate‐reader (BMG Labtech, Ortenberg, Germany, cat. no. 430–101) to evaluate hemolysis by the free hemoglobin content of the plasma.

### 2.4 Murine CSF

Mice were euthanized by intraperitoneal injection of pentobarbital (Apoteket AB, Solna, Sweden, cat. no. 338327; 600 mg/kg body weight). Once the animals exhibited no pain reflexes and had ceased breathing, a surgical incision was made at the base of the skull to expose the cisterna magna. A pulled glass capillary with a broken tip was carefully inserted through the dura mater into the cisterna magna, avoiding blood vessels and brain tissue. 3–5 μL CSF was collected, transferred into a prelabeled collection tube, immediately snap‐frozen in liquid nitrogen, and stored at −80°C. until analysis. CSF was pooled from two male nontransgenic C57Bl/6 mice (Charles River Laboratories, Wilmington, MA) both 212 days of age from our in‐house breeding of transgenic mice expressing different human SOD1s and from three male mice expressing wild‐type human SOD1 (line N1029) (Gurney et al. 1994) 470, 473, and 476 days of age, respectively. Transgenic mice are identified using an enzyme‐linked immunosorbent assay (ELISA) detecting human SOD1 (Zetterström et al. 2011). The use and maintenance of the mice and the experimental procedures described in this article were approved by the Regional Ethical Committee for Animal Research (dnr A20‐2023).

### 2.5 Homogenization of Tissues

Cervical and lumbar spinal cord ventral horns, brain gray matter from the precentral gyrus, and superior temporal gyrus as well as choroid plexus and the peripheral tissues liver, kidney, and gastrocnemius muscle were analyzed. Tissues samples were homogenized with an T25D Ultraturrax apparatus (IKA, Staufen, Germany, cat. no 0010004858) for 30 s and followed by pulsed sonication (1 s on/0.5 s off) during 1 min at 10% amplitude with samples on ice using a Branson Digital Sonifier SFX 250 with a 3 mm wide probe (Branson Sonifiers, Danbury, Connecticut, USA, Research Resource Identifier (RRID) SCR_024710) in 25 volumes of ice‐cold phosphate‐buffered saline (PBS, 10 mM K‐phosphate: KH_2_PO_4_ e, Barcelona, Spain, cat. no. PO0260/Na_2_HPO_4_ Merck, Darmstadt, Germany, cat. no. 1.06580 in 150 mM NaCl Duchefa Biochemie, Haarlem, the Netherlands, cat.no. S0520, pH 7.0) containing an antiproteolytic cocktail (Complete, Roche Diagnostics, Basel, Switzerland, cat. no. 1836145). The homogenates were stored at −80°C until analysis. Before analysis, the tissue homogenates were thawed at RT and sonicated for 1 min before dilutions were made for immunoblotting.

### 2.6 Antibodies

A set of eight antipeptide polyclonal antibodies was previously generated in rabbits immunized with synthetic peptides corresponding to amino acids 3–20, 24–39, 43–57, 57–72, 80–86, 100–115, 111–127, and 131–153 of human SOD1 (hSOD1). In combination, these peptides cover most of the sequence of hSOD1 and have been validated as being specific for unfolded SOD1 without binding to SOD1 with the native conformation (Bergh et al. 2015; Forsberg et al. 2011, 2010; Jonsson et al. 2004). Anti‐SOD1 antibodies were also raised in a goat using fully Cu‐ and Zn‐charged and natively folded hSOD1 purified from human erythrocytes (Marklund et al. 1997) as previously described (Zetterström et al. 2011). All procedures were approved by the Reginal Ethical Committee for Animal Research (dnrs A106‐1997, A46‐2007, and A87‐2011). Antibodies were custom‐made by Agrisera (Umeå, Sweden) and if not commercially available from Agrisera, may be shared upon reasonable request.

### 2.7 Immunoblotting

Western blotting was generally performed as previously described (Leykam et al. 2025). CSF samples, erythrocyte lysates, or tissue homogenates were diluted 1 + 1 with 2 × sample buffer for SDS‐PAGE containing 2‐mercaptoethanol (Sharlau, cat. no. ME00950250) and 10 μL of each sample were loaded on 26 well Criterion TGX Stain‐Free Any‐KD precast gels (Bio‐Rad, Hercules, California, USA, cat. no. 56781259) if not stated otherwise in the figure legend. The running buffer was from Bio‐Rad (cat. no. 1610772). The gels were run for 41 min at 200 V using a PowerPac Basic Power Supply (Bio‐Rad, cat. no. 1645050). Proteins were transferred to nitrocellulose membranes using Trans‐Blot Turbo Midi Nitrocellulose transfer packs (Bio‐Rad, cat. no. 1704159) and a Trans‐Blot Turbo Transfer System (Bio‐Rad, RRID SCR_023156) using the 7 min turbo protocol for midi gels. The membranes were blocked for 1 h at RT in 5% nonfat dry milk dissolved in 20 mM Tris‐buffered saline containing 0.1% Tween‐20 (TBST, TrizmaBase, Sigma Aldrich, St. Louis, Missouri, USA, cat. no. T1503 and Tween‐20, Sigma Aldrich, cat. no. P1379). The 24–39 antipeptide hSOD1 antibody was used at a concentration of 1 μg/mL and incubated over night at 5°C. Following washing with TBST, the filters were incubated for 60 min at room temperature with the secondary HRP‐conjugated polyclonal goat anti‐rabbit antibody (P0440 by DAKO, Santa Clara, California, USA, RRID AB2617138) at a dilution of 1:10000 and subsequently washed with TBST. The chemiluminescent reagent ECL Select (Cytiva, Marlborough, Massachusetts, USA, cat.no. RPN2235) and the ChemiDoc Touch Imager (Bio‐Rad, RRID SCR_019037) was used. Evaluation and quantification of the band intensities was done with the ImageLab software (Bio‐Rad, RRID SCR_014210). For the investigation of disulfide status of SOD1, CSF samples were diluted 1 + 1 with standard 2 × sample buffer containing 2‐mercaptoethanol for the reduced samples or 2 × sample buffer without 2‐mercaptoethanol but containing 20 mM iodoacetamide (IAM, GE Healthcare, Uppsala, Sweden, cat. no. RPW6302) for the nonreduced samples. After electrophoresis the gel was cut in half and one part was incubated 10 min at RT in transfer buffer (48 mM TrizmaBase, 39 mM glycine [Duchefa Biochemie, cat. no. G0709], and 0.37% SDS [Merck, cat. no. 18553]) containing 2% 2‐mercaptoethanol (in‐gel reduction) (Zetterstrom et al. 2007). The gel was washed twice for 5 min with transfer buffer without 2‐mercaptoethanol before being transferred to nitrocellulose membrane together with the other part of the gel. Uncropped western blots are shown in Figure S1 online.

### 2.8 N‐Terminal Protein Sequencing (Edman Degradation)

Fractions containing SOD1 after MonoQ separation (see below) of CSF from control individuals was concentrated by Amicon Ultra‐15 (Millipore, Burlington, MA, USA, cat. no. UFC901024) centrifugation units with a cellulose cut‐off filter of 10 kDa and separated by SDS PAGE using a 15% Tris–HCl gel (Bio‐rad, cat. no. 3450020). The proteins were transferred to PVDF membranes (GE Healthcare, cat. no. 106000023) and stained with Coomassie blue R‐250 (Fluka, Buchs, Switzerland, cat. no. 27815). Bands representing native and N‐truncated SOD1 were carefully excised with a clean scalpel and transferred to 1.5 mL tubes. Three rounds of samples were sent to Cambridge Peptides LTD (Cambridge, UK, www.cambridgepeptides.com) and one round of samples to Alphalyse (Odense, Denmark, www.alphalyse.com) for N‐terminal protein sequencing.

### 2.9 Size Exclusion Chromatography

Size exclusion chromatography (SEC) was performed as previously described (Zetterstrom et al. 2013). CSF from control individuals was pooled and 250 μL applied to a 1 cm × 30 cm Superdex 200 column (GE Healthcare, cat. no. 17‐5175‐01), eluted at 4°C with PBS at 45 mL/h, and collected in 0.3 mL fractions. The column was calibrated with Gel filtration calibration kits (GE Healthcare, cat. nos. 28‐4038‐41/42 and 17‐0441‐01) using blue dextran (2000 kDa), thyroglobulin (669 kDa), ferritin (440 kDa), aldolase (158 kDa), conalbumin (75 kDa), bovine serum albumin (BSA) (69 kDa), ovalbumin (44 kDa), SOD1 (32 kDa), and ribonuclease A (13.7 kDa). Fractions were stored at −80°C until analysis.

### 2.10 Anion‐Exchange Chromatography

Pools of CSF were made from control individuals. Around 35 mL of each pool were added to 10 mL Amicon Ultra (Millipore, cat. no. UFC801008) centrifugation units with a cellulose cut‐off filter of 10 kDa in three aliquots. After a 5 min centrifugation at 3500 × g in a Sigma 4 K15 centrifuge (Sigma Laborzentrifugen, Osterode am Harz, Germany), with the 11 156 swing out rotor (Sigma Laborzentrifugen), 10 mL of 20 mM Tris–HCl pH 7.5 were added, and the samples were recentrifuged. The centrifugation and buffer supplementation were repeated three additional times. The retentate after the last centrifugation was collected from the retention pocket and the volume adjusted to 10 mL with the Tris–HCl buffer. The sample was loaded onto a 1 mL 5/50 GL MonoQ anion‐exchange column (GE healthcare, cat. no. 17‐5166‐01) equilibrated with 20 mM Tris–HCl pH 7.5 using an ÄKTApurifier 10 chromatography system (GE Healthcare). After washing, bound proteins were eluted with a 30 mL linear gradient of 1 M NaCl in 20 mM Tris–HCl pH 7.5 ending at a concentration of 150 mM NaCl. The flow rate was 1.0 mL/min, fractions of 0.5 mL were collected, and absorbance at 280 nm was continuously recorded.

### 2.11 Coupling of Antibodies to M‐280 Tosyl‐Activated Dynabeads

The antibody raised in a goat against native hSOD1 were coupled to magnetic M‐280 tosyl‐activated Dynabeads (Thermo Fisher Scientific, Waltham, Massachusetts, USA, cat. no 14203) at a concentration of 20 μg antibody per mg beads according to the manufacturer's description. The binding buffer was 0.1 M borate pH 9.5 and 0.1 M borate containing 3 mM ammonium sulfate, and the antibody‐bead coupling was performed using an Intelli‐Mixer RM‐2 L (ELMI, Riga, Latvia, cat. no. 1SE021) agitating the samples at 10 rpm over night at 37°C. Binding efficiency was determined by absorbance at 280 nm measured with a DeVovix DS‐11 spectrophotometer (DeNovix, Wilmington, Delaware, USA, cat. no. DS‐11). The beads were washed once with 1 mL of washing buffer 1 (10 mM sodium phosphate buffer [JT Baker, Radnor, PA, USA, cat. no. O303] pH 7.4 containing 150 mM sodium chloride and 1% BSA IgG1 free [Jackson ImmunoResearch, Cambridgeshire, United Kingdom, cat. no. 001‐000‐162]) and two times with washing buffer 2 (10 mM sodium phosphate buffer pH 7.4 containing 150 mM sodium chloride and 1% BSA IgG1 free with 0.5% NP‐40 [Millipore, cat. no 492016]) and stored at 4°C in washing buffer 2 until use at a concentration of 8.3 mg beads per mL buffer.

### 2.12 Immunocapture of SOD1 Protein

Bead solution (10 μL) containing 83 ng Dynabeads carrying 1.66 ng antibody was transferred to 1.5 mL tubes and washed twice with 500 μL binding buffer (PBS, pH 7.4 with additional 0.15 M sodium chloride, 0.5% BSA IgG1 free and 80 μL/mL Complete EDTA‐free). The tubes were vortexed at 12000 rpm for 15 s, incubated for 2.5 min, placed on a magnetic holder for 2 min and the supernatants were removed. Next, 50 μL CSF diluted 1:2 with binding buffer was added to the antibody beads. After mixing the solution by vortexing at 12000 rpm for 15 s, the tubes were placed onto an Intelli‐Mixer RM‐2 L and incubated for 15 min in RT using the shaking program U at 60 rpm. The tubes were then placed on the magnetic holder and after 2.5 min the sample solution was transferred into a fresh tube for western blot analysis (nonbinding sample). Washing of the beads was done in three consecutive steps, once with 100 μL and twice with 500 μL washing buffer (PBS, pH 7.4 with additional 0.15 M sodium chloride). Samples were vortexed at 12000 rpm for 15 s, incubated for 2.5 min at RT without mixing before being placed on a magnetic holder for 2 min, and the buffer was removed. The 100 μL washing buffer of the first step was saved for later analysis. Elution of the captured SOD1 was performed by adding 50 μL 0.1 M glycine buffer, pH 3. The samples were resuspended at 12000 rpm, incubated for 4 min at RT and placed for 1 min on the magnet. The solution was then immediately transferred to a sample collection tube containing 1.25 μL 1 M Tris–HCl buffer, pH 9 for neutralization of the sample. Collection tubes were again vortexed at 12000 rpm for 15 s. The different samples were then analyzed by western blot. The CSF sample before immunocapture and the nonbinding sample were also analyzed by mass spectrometry. SOD1 was also immunocaptured from blood plasma using the same protocol with the following exceptions: 20 μL bead solution was used per sample, the incubation time was 60 min, the sample size was 500 μL plasma, and bound protein was eluted by boiling in 60 μL 1 × sample buffer for western blot resulting in a 8.3 times concentration of the sample.

### 2.13 Isolation of SOD1 by Molecular Weight Cut‐Off Centrifugation

Five hundred microliter of CSF pooled from control subjects without neurodegenerative diseases were transferred into Amicon Ultra 0.5‐mL cellulose centrifugation units (Millipore, cat. no. UFC501096) with 10 kDa molecular weight cut‐off. The CSF samples were centrifuged at 14000 × g for 30 min at RT and the flow‐through was saved for later analysis. Proteins retained on the filter were collected by reverse spin at 1000 × *g* for 2 min at RT. The collected retentate was brought to a final volume of 500 μL using PBS and frozen at −80°C. The flow‐through and the retentate were analyzed by mass spectrometry or diluted 1:2 with 2 × western blot sample buffer containing 2‐mercaptoethanol for western blot analysis.

### 2.14 Mass Spectrometry

Quantification of N‐acetylated and Cys‐carbamidomethylated SOD1 1–26 (Ac‐ATKAVCVLKGDGPVQGIINFEQKESN) was performed by parallel reaction monitoring (PRM) using a nano‐LC (Ultimate RSLC Nano, Thermo Fisher Scientific) equipped with a C_18_ trap and separation columns (PepMap Acclaim 300 μm × 5 mm, Thermo Fisher Scientific, PepMap Acclaim 75 μm × 500 mm, Thermo Fisher Scientific, respectively) coupled to a Fusion Tribrid Orbitrap mass spectrometer equipped with an Easy Spray nano‐ESI source, and fitted with a high field asymmetric waveform ion mobility spectrometry device (all from Thermo Fisher Scientific). The mobile phase consisted of 0.1% formic acid (FA; Thermo Fisher Scientific, cat. no. 28905) (Buffer A) and 84% acetonitrile (ACN, HPLC grade, cat. no. 042880.K2), 0.1% FA (Buffer B). Peptide separation was performed using a linear gradient of 4%–40% Buffer B over 40 min, followed by washing and equilibration. The FAIMS device was operated in the standard resolution mode, using a carrier gas flow of 0.7 L/min and compensation voltage −50 V. Targeted MS/MS spectra were recorded in the Orbitrap of the triply‐charged peptide ion (m/z 948.8235) and its heavy‐labeled analog (m/z 951.4949), using 1.6 m/z quadrupole isolation window, 120 000 resolution, 246 ms ion injection time, 500% AGC target, 29% HCD collision energy. Samples were spiked with 25 fmol synthetic peptide standard (AQUA basic, Thermo Fisher Scientific), labeled with ^13^C_6_^15^N_2_ at Lys‐3, and quantification was performed using the heavy‐to‐light peptide ratio. Data processing was performed using Skyline (https://skyline.ms/project/home/begin.view).

### 2.15 MisELISA

ELISAs that specifically detect misfolded SOD1 have previously been developed in our laboratory (misELISA) (Zetterström et al. 2011). Here we used the misELISA based on antibodies targeting amino acids 57–72 in hSOD1 as the capturing antibody. This antibody reacts only with highly disordered SOD1 species and lacks affinity for the natively folded protein (Bergh et al. 2015; Forsberg et al. 2011, 2010). The secondary antibody was raised in a goat against denatured SOD1. This antibody reacts preferentially with unfolded SOD1 (Zetterström et al. 2011). The misELISA was calibrated with a spinal cord homogenate from a transgenic mouse expressing the G127X mutation in SOD1. One unit of disordered SOD1 is defined as the amount present in 1 g wet weight of the G127X standard (Zetterström et al. 2011).

### 2.16 Statistics

Quantitative data are generally presented as mean ± standard deviation (SD). Statistical analyses were performed using SPSS software (version 29; SPSS Inc., Chicago, IL, USA; RRID:SCR_002865). Given the limited sample size, no formal outlier testing was conducted; instead, all observations were retained for analysis. An independent two‐sided *t*‐test was applied, as the data were normally distributed (Shapiro–Wilk test) and the test is considered robust for small sample sizes, minimizing the risk of overestimating differences. A priori sample size calculation was not feasible for N‐terminally truncated SOD1 levels in samples with high or low misfolded SOD1, as this study represents the first attempt to estimate the effect size. Statistical significance was set at *α* = 0.05. A complete statistical summary is provided as Table S1.

## 3 Results

### 3.1 Low Molecular‐Weight SOD1 in CSF

**FIGURE 1:**
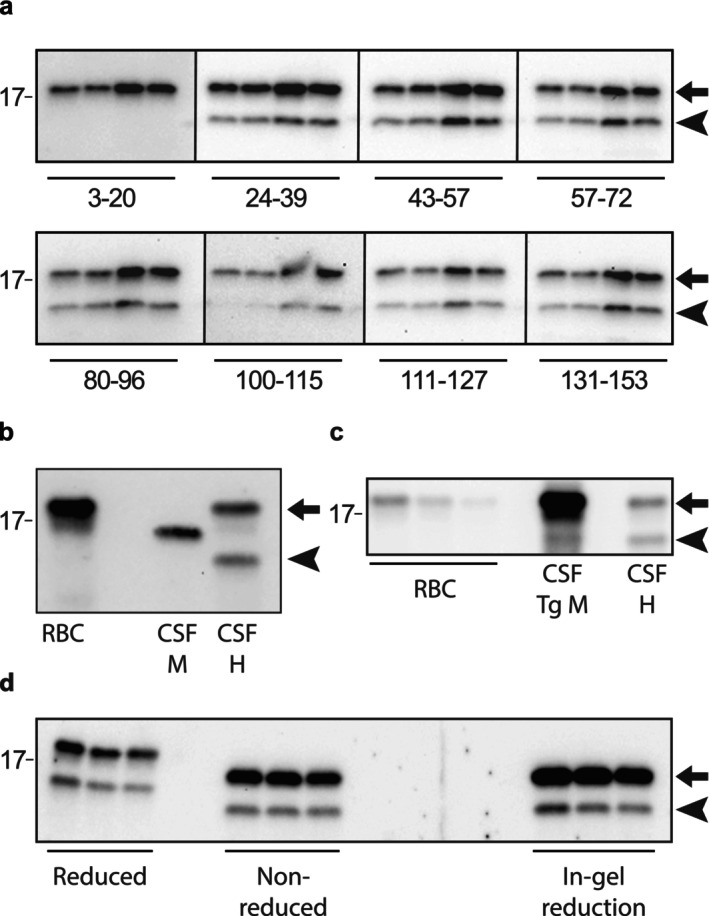
Western blot showing N‐truncated SOD1. (a) Western blot analysis of four pooled CSF samples using eight anti‐SOD1 peptide antibodies spanning the full protein sequence. The upper band of 18 kDa represents full‐length SOD1 (marked with an arrow), while the lower band of ~15 kDa (marked with an arrowhead) represents the N‐terminally truncated SOD1 species. 12.5% Criterion precast Tris–HCl gels were used. (b) Western blot of murine CSF from nontransgenic C57Bl/6 mice shows no evidence of truncated SOD1 (CSF M). A human hemolysate (RBC) and human CSF (CSF H) are also shown. No band corresponding to N‐truncated SOD1 is visible in human erythrocytes, nor in murine CSF. The anti 57–72 antibody was used due to almost identical sequence of human and murine SOD1 in this region, and it binds both forms of SOD1. (c) CSF collected from three transgenic mice expressing wild‐type human SOD1 was pooled and subjected to western blot to investigate if human SOD1 becomes truncated in a different species (CSF Tg M). Dilutions of a human hemolysate (RBC) and human CSF (CSF H) are also shown. Only a small proportion (< 3%) of the human SOD1 in transgenic mice was found to be truncated. An antibody raised against amino acids 24–39 in human SOD1 was used since it only detects human SOD1 because the human and murine SOD1 sequence are not conserved in this region. (d) Three independent pools of CSF samples from control individuals were analyzed by western blot under reducing conditions and nonreducing conditions (omitting 2‐mercaptoethanol from sample buffer) with or without in‐gel reduction. See Material and Methods for detailed experimental procedures. Both full length and N‐terminally truncated SOD1 migrated further under nonreducing conditions. No extra bands representing nonnative disulfide bonds were seen.

A previous study revealed a distinct band with higher electrophoretic mobility than native SOD1 on western blots of human CSF samples stained by anti‐SOD1 antibodies. The position indicated that the band represented a SOD1 fragment ~3 kDa smaller than the native subunit. Western blots using seven different anti‐SOD1 peptide antibodies, together covering most of the SOD1 sequence, showed that all but the antibody targeting amino acids 3 to 20 of human SOD1 bind to the SOD1 fragment. These results support the interpretation that the observed band corresponds to an N‐terminally truncated SOD1 variant protein present in the analyzed CSF samples from ALS patients and controls (Jacobsson et al. 2001). To validate these findings, we analyzed four pooled CSF samples collected from patients referred to the neurology department at Umeå University Hospital for nonneurodegenerative causes with western blot using an array of eight SOD1‐specific antipeptide antibodies. The results confirmed our findings in the previous study (Figure 1a). Only the antibody directed against amino acids 3 to 20 in the N‐terminus of SOD1 did not detect the lower molecular weight band. Since both the native band and the band with lower molecular weight are visible using the other antibodies, both forms occur simultaneously in these CSF pools. Also, in samples from single individuals, both controls and ALS patients, both SOD1 variants are seen, and the N‐truncated form is always accompanied by the normal subunit (Leykam et al. 2024). Quantification showed that the N‐terminally truncated SOD1 variant constituted 34% ± 3% (mean ± SD, *n* = 24) of the total SOD1 content in control individuals in good agreement with previous findings (Jacobsson et al. 2001). Attempts to detect the cleaved N‐terminal peptide by western blotting optimized for small proteins were unsuccessful (not shown).

To assess cross‐species occurrence of truncated SOD1, murine CSF was analyzed by western blot together with a human hemolysate and CSF sample to benchmark the different SOD1 species. No truncated form of the endogenous murine SOD1 protein could be detected in the CSF (Figure 1b). Note that murine SOD1 has a higher mobility than human SOD1. To investigate if human SOD1 expressed in mice is truncated in the same way as in humans, CSF was collected from transgenic mice expressing high levels of wild‐type human SOD1 (Gurney et al. 1994). Western blot using an antibody specific for human SOD1 could identify the truncated human SOD1 variant also in CSF from transgenic mice (Figure 1c), however, in very low concentration. Densiometric analysis of the western blot showed that < 3% of the human SOD1 carried the truncation.

A critical determinant of SOD1 stability is the intramolecular disulfide bond between Cys57 and Cys146 (Jonsson et al. 2006; Zetterstrom et al. 2013, 2007). To assess the disulfide status of SOD1 in cerebrospinal fluid (CSF), nonreducing western blot analyses were performed in the presence of IAM to prevent disulfide exchange during sample heating prior to electrophoresis. Postelectrophoretic reduction (in‐gel reduction), which can enhance antigen detection, was also employed to improve the likelihood of identifying low‐abundance variants carrying aberrant, nonnative disulfides (Zetterstrom et al. 2013, 2007). Both native and N‐terminally truncated SOD1 species in CSF from control individuals exhibited faster migration when reductant was omitted from the sample buffer (Figure 1d). The identical shift observed for both forms suggests that each retains the native Cys57‐Cys146 disulfide bond. No additional bands corresponding to nonnative or reduced SOD1 species were detected, even following in‐gel reduction, indicating that all SOD1 present in CSF is fully oxidized (Figure 1d).

### 3.2 Low Molecular‐Weight SOD1 in CSF Is N‐Truncated

**FIGURE 2:**
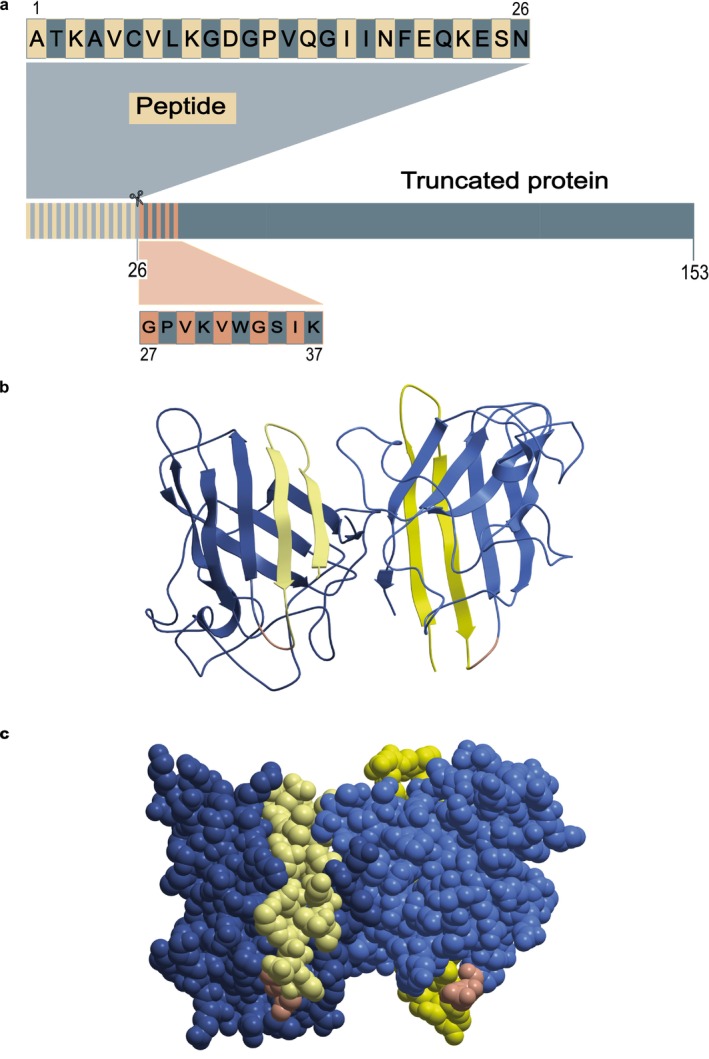
Truncation site in SOD1. (a) Illustration of the SOD1 amino acid sequence with the identified truncation site. With Edman degradation the first 10 amino acids (orange) of the truncated protein could be sequenced (27–37 in full‐length SOD1). The identified sequence is identical to the amino acids of native SOD1 protein from glycine 27 and forward. Hence, the truncation site is positioned between amino acids Asn‐26 and Gly‐27. The sequence of the cleaved peptide in wildtype SOD1 is shown (yellow). (b) The crystal structure of wildtype holo SOD1 (PDB 1HL5, (Strange et al. 2003)) was visualized with the MolsoftBrowser64 version 3.9–4. (Molsoft L.L.C, La Jolla, California, USA). The two SOD1 subunits are colored in blue. The N‐terminal 26 amino acids representing the short peptide fragment are colored in yellow. A ribbon representation shows that the N‐terminal part of SOD1 partly forms the dimer interface and that the cleavage site (orange) is easily accessible at one pole of the monomer. (c) Space filling model showing that the N‐terminal most part of SOD1 is tightly bound to the remaining subunit as well as to the other subunit. The cleaved N‐terminal peptide is highlighted in yellow, the remaining subunit in blue, and the site of truncation in orange.

As shown above, the N‐terminally truncated SOD1 band is reactive with seven different SOD1 antibodies, but not with an N‐terminal antibody having the epitope SOD1 3–20. We therefore aimed to identify the exact position of the cleavage and utilized N‐terminal protein sequencing (Edman degradation). In this method, the N‐terminal residues of a peptide are sequentially cleaved off and identified by HPLC analysis, allowing identification of the amino acid sequence from the N‐terminal end, which in this case will identify the endogenous cleavage site in SOD1. Proteins in a pooled CSF sample were separated by SDS PAGE, blotted onto a PVDF membrane, stained with Coomassie R‐250, and the bands corresponding to both the native and N‐truncated SOD1 variants were excised and sent for sequencing. The upper band could not be N‐terminally sequenced, probably due to N‐terminal acetylation, a posttranslational modification known to be present in SOD1 (Barra et al. 1980) that effectively blocks the Edman method. The lower band could however be sequenced, and the amino acid sequence is illustrated in Figure 2. The N‐terminus of this protein is identical to the sequence from position 27 to 37 in human SOD1 showing that the protein is an N‐terminally truncated SOD1 variant cleaved between the residues asparagine 26 and glycine 27 leaving a cleaved peptide of 26 amino acids. The 26 most N‐terminal amino acids of wildtype SOD1 are ATKAVCVLKGDGPVQGIINFEQKESN (after removal of the starting M). The theoretical molecular weight of this peptide is 2.6 kDa in good agreement with the western blot for where the truncated SOD1 variant appears around 3 kDa below the molecular weight of the wildtype SOD1 monomer (Figure 1).

### 3.3 N‐Truncated SOD1 Variant Is Abundant in CSF but Not CNS Tissue

**FIGURE 3:**
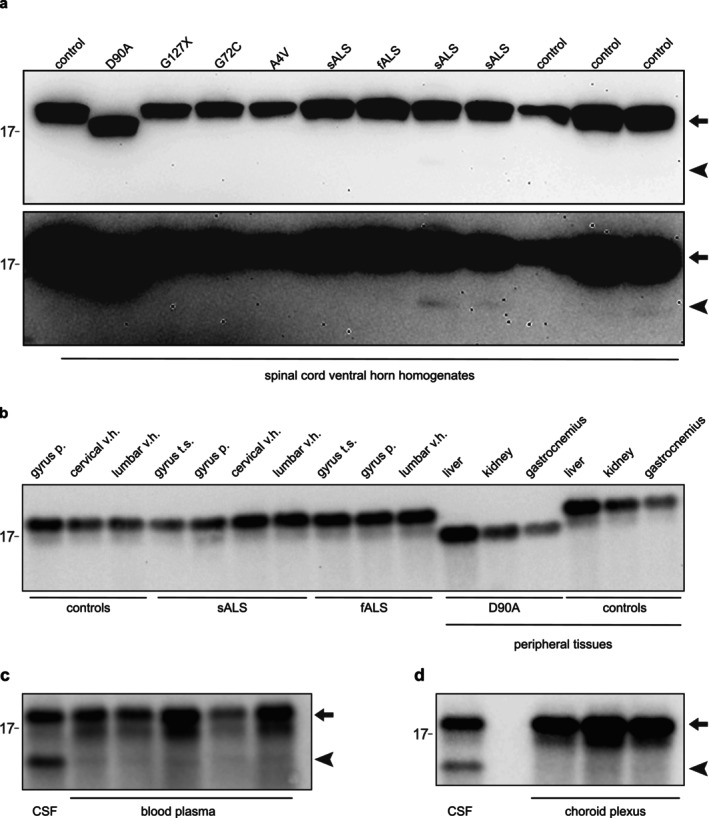
Western blot analysis of CNS and peripheral tissue homogenates, and plasma, showing minimal presence of truncated SOD1 compared to CSF. The upper band of 18 kDa represents full‐length SOD1 (marked with an arrow), while the lower band of ~15 kDa (marked with an arrowhead) represents the N‐terminally truncated SOD1 species. (a) Western blot of spinal cord ventral horn homogenates from ALS patients and control postmortem tissues. SOD1 mutation is stated if present. Long and short exposure is shown. (b) Western blot of postmortem tissues obtained from ALS patients and controls covering the central nervous system as well as the peripheral tissues liver, kidney and gastrocnemius muscle. (c) SOD1 immunocaptured from EDTA plasma. A CSF sample is shown for comparison. (d) Tissue homogenates of choroid plexus from three control individuals. A CSF sample is shown for comparison. None or only faint bands of a truncated SOD1 variant is discernable in CNS, including choroid plexus, and in peripheral tissues and blood plasma.

The CSF reflects the situation of the CNS and is partly formed by secretion in the choroid plexus, partly from CNS extracellular fluid containing secreted proteins and unspecifically leaked protein, as well as proteins released from injured or dying cells (Wichmann et al. 2021). The truncated SOD1 found in CSF might therefore originate from CNS parenchyma. To investigate if the N‐truncated subunit is present in different areas of the central nervous system, we immunoblotted homogenates of postmortem samples from the spinal cord ventral and dorsal horns, motor cortex, temporal lobe, and choroid plexus as well as the peripheral tissues liver, kidney, and skeletal muscle from controls and ALS patients. Trace amounts of a lower molecular weight band, consistent with truncated SOD1, were detected as observed in CSF in some samples from the CNS but also in peripheral tissues. However, the levels were very low compared to CSF and represented < 0.1% of the total SOD1 (Figure 3).

We also analyzed blood plasma, an extracellular fluid like CSF. The total level of SOD1 in blood plasma is about 10% of the CSF levels (Marklund et al. 1986). Normal vacuum collection of venous blood will cause hemolysis and complicate analysis of SOD1 in the blood plasma. Blood was therefore collected by spontaneous flow through 18G needles to reduce hemolysis from five healthy anonymous donors. Analysis of free hemoglobin showed that some hemolysis had occurred, and the levels would indicate that about 1/3 of the SOD1 in the plasma derived from lysed erythrocytes (not shown). However, erythrocytes do not contain any N‐truncated SOD1 (Figure 1b). The plasma samples had to be concentrated by immunocapture using an antibody that binds native and nonnative forms of SOD1 to be visible by western blotting. The plasma samples did contain small amounts of truncated SOD1 (Figure 3c), but far less than is seen in CSF.

### 3.4 Biochemical Characterization of the N‐Truncated SOD1

**FIGURE 4:**
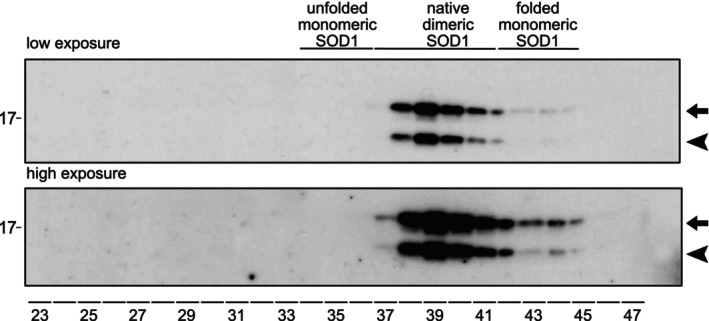
Size exclusion chromatography of SOD1 in CSF. Western blot of SEC fractions 23–47 from pooled CSF, showing coelution of full‐length and truncated SOD1 in native dimer range. The 24–39 SOD1‐specific antipeptide antibody was used. Long and short exposure is shown. No SOD1 was found in the void volume (fractions 23–24). The upper band of 18 kDa represents full‐length SOD1 (marked with an arrow), while the lower band of ~15 kDa (marked with an arrowhead) represents the novel N‐terminally truncated SOD1 species. A 12.5% Criterion precast Tris–HCl gel was used.

To evaluate the effect of the N‐terminal truncation on the folding of SOD1, CSF from two neurological controls were evaluated by size exclusion chromatography on a Superdex‐25 column. Both full‐length and truncated SOD1 eluted in fractions 37–45 (Figure 4), consistent with native dimeric conformation (Zetterstrom et al. 2013, 2007). No high‐molecular weight forms of SOD1 could be detected in the void volume (Figure 4). Folded monomeric SOD1 normally elutes in fractions 42–44 and disordered monomeric SOD1 elutes in fractions 34–36 in this column (Zetterstrom et al. 2013). No evidence for monomeric SOD1 was found in any of these positions in the chromatograms (Figure 4). The peaks for the truncated and normal subunits were found in the same fraction with almost identical elution profiles. There is thus no evidence for SOD1 present with a hydrodynamic radius deviating from that of native SOD1, suggesting that the truncated variant is incorporated into the normal dimers. Hence both fragments of the truncated SOD1 seem to stay in their normal positions in the structure of the native dimer.

**FIGURE 5:**
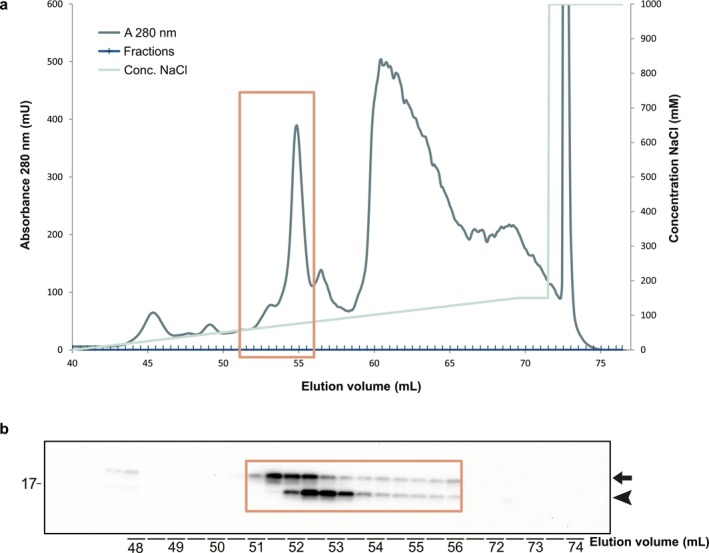
Anion exchange chromatogram of CSF showing separation of full‐length and truncated SOD1 based on surface charge. (a) Chromatogram from separation of a CSF samples with a MonoQ anion exchange column. Protein absorbance measured with A280 as well as the NaCl gradient used for elution are shown. (b) Fractions of 500 μL were collected and fractions containing 48–56.5 mL as well as 72–74 mL were analyzed with western blot using the 24–39 anti‐SOD1 antibody. The square indicates which fractions from the chromatogram were blotted. The upper band of 18 kDa represents full‐length SOD1 (marked with an arrow), while the lower band of ~15 kDa (marked with an arrowhead) represents the N‐terminally truncated SOD1 species. No SOD1 was found in fractions eluted with high NaCl concentration (72–74 mL).

An efficient way to purify SOD1 is by anionic exchange chromatography that can separate SOD1 variants with a single charge difference (Marklund et al. 1997). Hence, the surface charge of SOD1 might be used to separate the truncated from full‐length SOD1 since several potentially charged amino acids are present in the truncated peptide. Anion exchange chromatography using a Mono‐Q column was used to separate N‐truncated from the native SOD1 (Figure 5). Native full‐length SOD1 eluted earlier than truncated variants, suggesting subtle charge differences with a slightly more negative surface charge of the truncated SOD1 subunits. In certain fractions, N‐terminally truncated SOD1 was more abundant than the native form, indicating the presence of homodimers composed solely of truncated subunits.

### 3.5 Peptidomic Identification of the N‐Terminal Peptide From Folded SOD1

Previous data shows that SOD1 found in CSF has a high specific activity and hence should maintain its native conformation (Jacobsson et al. 2001). Even though the most N‐terminal amino acids in SOD1 are not involved in the formation of the active site and the substrate directing electrostatic loop, they play a crucial role for the stability and activity of the folded protein. The loop structures connecting the β‐barrel sequences together with hydrogen bond bridges between loop side chains and main chain maintain the overall conformational framework (Parge et al. 1992). For SOD1 to be active, the complete protein is needed and C‐terminally truncated SOD1s have shown a total lack of enzymatic activity (Jonsson et al. 2004; Keskin et al. 2017). For the truncated SOD1 to be active the cleaved short and long peptides must stay in their native positions in relation to the remaining subunit in the native fold. Size exclusion chromatography with truncated and native SOD1 eluting in the same fractions supports this hypothesis (Figure 5). Three‐dimensional representations of the native SOD1 structure (Figure 2) also shows that the N‐terminal peptide forms β‐strands one and two who are tightly bound to β‐strands eight and three to form the β‐barrel. The cleavage site is easily accessible for a peptidase on either side of the molecule. This indicates that the structure is rigid and could stay folded even after cleavage of the peptide backbone between asparagine‐26 and glycine‐27.

**FIGURE 6:**
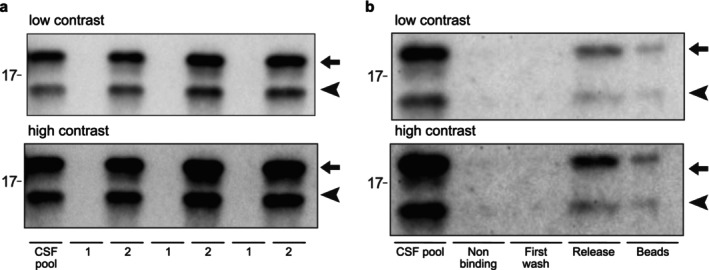
Comparison of SOD1 isolation by molecular weight filtration and immunocapture, confirming peptide retention with the SOD1 protein. The upper band of 18 kDa represents full‐length SOD1 (marked with an arrow), and the lower band of ~15 kDa (marked with an arrowhead) represents the N‐terminally truncated SOD1 species. (a) CSF samples were centrifuged through a 10 kDa cut off filter. 1 denotes the fraction of the sampled that passed the filter and 2 denotes the fraction that was retained on the filter. The SOD1 dimer is 32 kDa and should be retained but the short peptide should pass. Three different samples are shown. (b) Immunocapture with a SOD1 specific antibody. Shown are the CSF pool used for immunocapture; nonbinding denotes the sample after 15 min of binding to the antibodies and removal of the antibody beads with bound protein. All SOD1 is captured on the beads. Next, the beads are washed with buffer and First wash shows that no SOD1 was released by the first washing step. The SOD1 that is bound to the beads is then released by boiling in SDS PAGE sample buffer and shown in the Release lane. Lastly the remaining beads are also loaded on the gel to control for incomplete release (lane marked Beads). Both native and truncated SOD1 are captured in the experiment.

To further verify that the peptide is bound to the remaining subunit, we first separated three different pooled CSF samples by molecular weight using a 10 kDa cut off centrifugation filter. Both the native and the N‐terminally truncated SOD1 variants should be captured on the filter but the short peptide should not. Western blot analysis revealed that both the truncated and the full‐length SOD1 variant were fully captured by the filter in all three samples and no SOD1 was found in the sample that passed the filter (Figure 6a). The peptide might conceivably be bound to some other component in the CSF samples and consequently not pass the filter. Therefore, we also used a complementing strategy with immunocapture of SOD1 by an antibody that binds both native and denatured SOD1 (Leykam et al. 2024). Western blot confirmed complete immunocapture of both full‐length and truncated SOD1 from CSF (Figure 6b).

**FIGURE 7:**
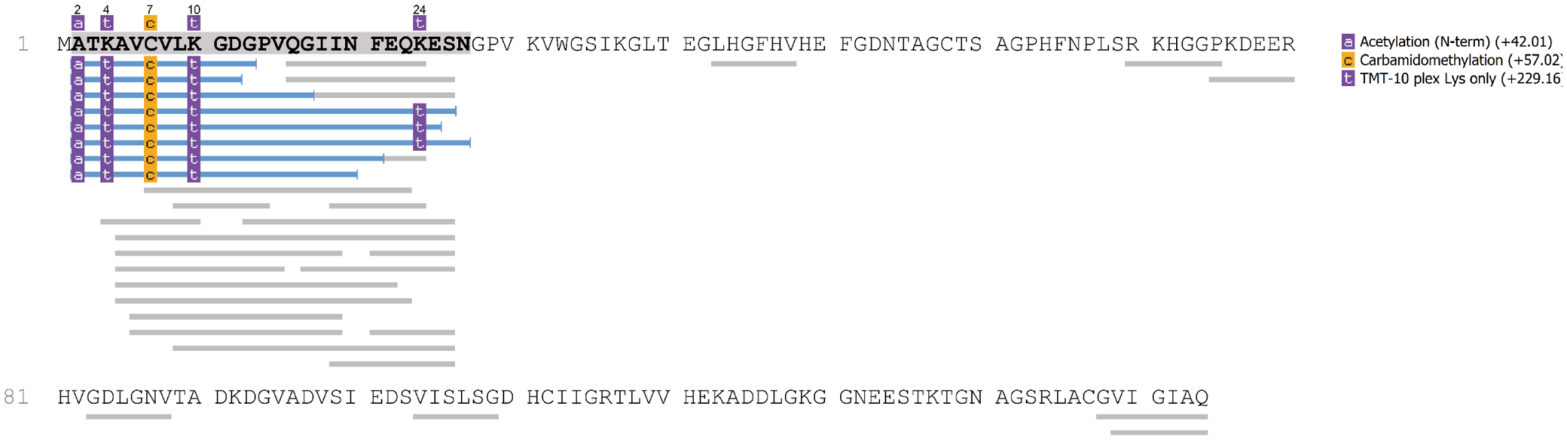
Proteomic identification of the short SOD1 peptide. Mass spectrometric identification of endogenous SOD1 peptides in CSF, predominantly from the N‐terminal region. Peptides represented by blue color were significantly detected in the CSF samples while peptides in gray did not reach the limit for FDR that was set to 1%. All identified peptides were from the N‐terminus with the longest peptide ranging from 2 to 26.

To search for the N‐terminal SOD1 peptide, data from a previous explorative peptidomics study (Skillbäck et al. 2017), in which CSF peptides were isolated by 30‐kDa MWCO ultrafiltration, labeled with tandem mass tag (TMT) reagents and analyzed by LC–MS were used. The mass spectrometry data were reinterrogated to search for SOD1 fragments, using automated de novo peptide sequencing (PEAKS X, Bioinformatics Solutions). Eight endogenous peptides were identified with a false discovery rate (FDR) below 1% (highlighted in blue), all containing the acetylated N‐terminal alanine residue. The longest peptide extended to Asn‐26, while the most abundant fragment terminated at Ser‐25 (Figure 7). These findings align with the cleavage site determined by Edman degradation and a release of the 1–26 peptide that is subsequently trimmed by exopeptidases from the C‐terminal end. Nearly all the less abundant SOD1 peptides (gray) also derived from the N‐terminal end.

To investigate whether this peptide remains attached to the SOD1 subunit in CSF, to maintain an intact quaternary protein structure, we developed a targeted mass spectrometric parallel reaction monitoring assay, with quantification by isotope dilution using a stable isotope labeled peptide analog. Mass spectrometry confirmed the presence of the N‐terminal peptide in the samples retained on the 10 kDa cut‐off filter and the immunocaptured samples (Table 1). The N‐terminal peptide was, however, not identified in the filtrate and only in minute amounts in the CSF sample after immunocapture, confirming that the peptide stays attached to the truncated and/or full‐length subunit (Table 1). The peptide ratio relative to standard was higher after immunocapture compared to after filtration, indicating that some of the sample may have been trapped by the filter.

**TABLE 1:** N‐terminal SOD1 peptide in CSF identified with mass spectrometry.

CSF sample	Peptide ratio to standard
CSF before immunocapture^*^	9.1 ± 3.6
CSF after immunocapture^+^	0.31 ± 0.12
Sample passed through filter^#^	0
Sample retained by filter^§^	2.6 ± 0.66

*Note:* N‐terminal peptide identified before (*) and after (+) immunocapture of SOD1. N‐terminal peptide identified before (#) and after (§) CSF was passed over a 10 kDa cut‐off filter. All samples analyzed in triplicates.

### 3.6 SOD1 in CSF Is Not Further Cleaved Ex Vivo

**FIGURE 8:**
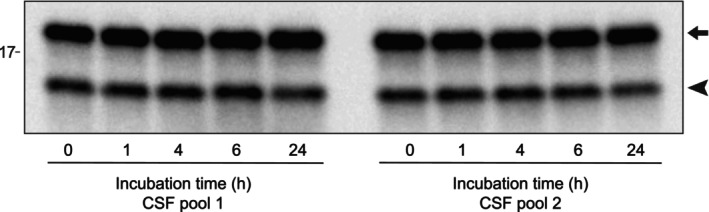
Western blot analysis after CSF incubation at 37°C shows no evidence of further N‐terminal proteolysis. Two pools of CSF were incubated at 37°C and samples for western blot taken after the indicated times. No change in the relative abundance of full‐length and truncated SOD1 was observed during incubation. The upper band of 18 kDa represents full‐length SOD1 (marked with an arrow), and the lower band of ~15 kDa (marked with an arrowhead) represents the N‐terminally truncated SOD1 species.

Where and how SOD1 is N‐terminally cleaved is not clear. We did not find increased levels of the N‐terminally cleaved form in the choroid plexus, nor in the other regions of CNS investigated (Figure 3). One possibility is that SOD1 is cleaved by an endopeptidase present in the CSF. If so, the process is likely to continue in vitro after sampling. To test for this possibility, we incubated two pools of CSF from control individuals at 37°C for 24 h with samples taken berfore incubation and at 1, 4, 6, and 24 h. Note that CSF turns over four times during a 24 h period. The samples were then analyzed by western blot. The absolute amounts or ratio between the full‐length SOD1 and the N‐terminally truncated variant did not change during this incubation indicating that SOD1 was not further cleaved ex vivo (Figure 8).

### 3.7 N‐Truncation Is Not Correlated With Misfolding of SOD1 in CSF

Inclusions of aggregated SOD1 in motor neurons are seen as a common hallmark of SOD1‐ linked ALS (Kato et al. 2000). The common evidence suggests that these ALS associated SOD1 aggregates are formed following a prion‐like templated‐assisted manner (Ayers et al. 2014; Bidhendi et al. 2016; Ekhtiari Bidhendi et al. 2018) similar to the one observed for the prion protein PrP^c^. The initial step in this process is the misfolding of the native protein and the N‐truncation could lead to increased SOD1 misfolding.

**FIGURE 9:**
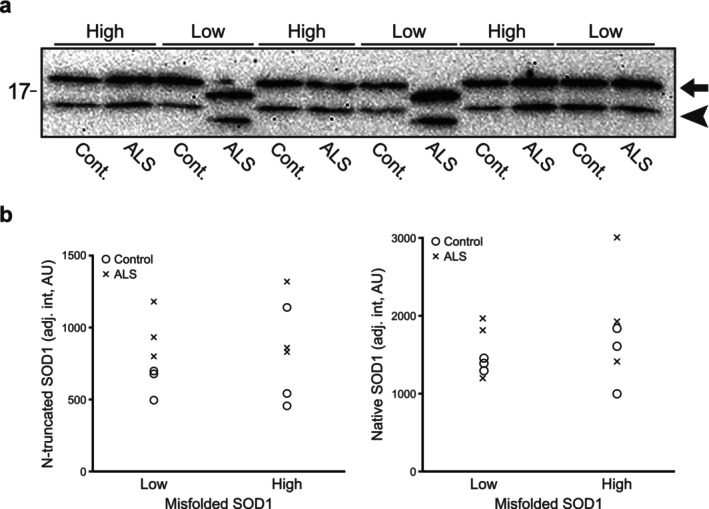
Western blot comparison of CSF from ALS patients and controls with varying levels of misfolded SOD1; truncated SOD1 levels remained unchanged. (a) CSF samples from ALS patients (*n* = 6) and controls (*n* = 6) with low (3 ALS patients and 3 controls) or high levels (3 ALS patients and 3 controls) of misfolded SOD1 were analyzed by western blots. The upper band of 18 kDa represents full‐length SOD1 (marked with an arrow), while the lower band of ~15 kDa (marked with an arrowhead) represents the novel N‐terminally truncated SOD1 species. Note that the D90A SOD1 variant has a faster migration on western blots. A 12.5% Criterion precast Tris–HCl gel was used. (b) Quantification of the bands showed that there was no difference in the amounts of the N‐truncated SOD1 nor the native full‐length protein in the samples with low or high levels of misfolded SOD1 (*p* > 0.05, two‐tailed independent samples *t*‐test, for a full statistical report, Table S1).

We have previously measured the levels of misfolded SOD1 in CSF by an ELISA selective for misfolded SOD1 called misELISA (Zetterström et al. 2011). To determine if the N‐terminally truncated SOD1 is involved in the misfolding of SOD1, we used CSF samples from persons with high levels of misfolded SOD1 and compared them to CSF samples containing low levels of misfolded SOD1 and measured the amount of N‐terminally truncated SOD1 in these samples. Three patients and three controls with low levels of misfolded SOD1 were compared to three patients and three controls with high levels of misfolded SOD1 by western blot. Overall, ALS patients and controls have the same levels of misfolded SOD1 in CSF (Zetterström et al. 2011) as was the case for this small subset: 3.6 ± 1.7 and 4.7 ± 2.6 μU/mL (mean ± SD) for ALS patients and controls, respectively. Western blot analysis revealed no correlation between misfolded SOD1 levels and abundance of truncated SOD1. Quantification of the N‐truncated band in samples with high levels of misfolding yielded an adjusted intensity of 860 ± 330 arbitrary units (AU), whereas samples with low misfolding showed 800 ± 240 AU. The difference was not statistically significant (*p* > 0.05, two‐tailed independent samples *t*‐test; Figure 9b). Quantification of the native band produced similar results (Table S1). This was a pilot experiment without a preceding power calculation. No physiologically meaningful standardized difference between groups to support a formal power calculation for further experiments was detected. These findings suggest that factors other than the identified N‐truncated SOD1 variant are driving SOD1 unfolding, and a larger study is unlikely to alter this conclusion regardless of sample size. However, the level of truncated SOD1 appeared elevated in these six ALS patients compared to the controls (Figure 9b).

The disordered SOD1 detected by misELISA accounts for about 1/1400 of the total SOD1 in human CSF (Zetterström et al. 2011). In disordered SOD1, the N‐terminal peptide is likely to have separated from the longer C‐terminal segment. This might explain the preponderance of SOD1 peptides in CSF derived from the 1–26 sequence (Figure 7).

## 4 Discussion

There are three different isoforms of SOD expressed in humans. The detection of a distinct band recognized by multiple anti‐SOD1 antibodies prompted investigation into its molecular identity (Jacobsson et al. 2001). It was hypothesized that it was an N‐truncated variant of SOD1. Our findings confirm this hypothesis and have identified the cleavage sites by mass spectrometry and Edman sequencing.

N‐terminally truncated SOD1 was detected in CSF and, to a lesser extent, in postmortem CNS tissues. The choroid plexus shows similarly low levels indicating that the truncated SOD1 is not specifically formed during secretion of the CSF. The absence of further cleavage during in vitro incubation suggests that truncation occurs in vivo. Instead, it indicates in situ cleavage in the parenchyma and subsequent release into the CSF or even preferential secretion into the extracellular space. However, the cleavage may still occur in the CSF compartment but only in vivo by for example, a surface‐bound peptidase. Our data indicate that both fragments of the N‐truncated SOD1 remain associated within a folded dimer, as dimers containing truncated SOD1 cannot be separated from native homodimers by size‐exclusion chromatography (Figure 4). Dimers incorporating truncated subunits must retain full enzymatic activity, given that SOD1 in CSF exhibits a specific activity of 4.10 U/ng (Leykam et al. 2024), which is comparable to that of fully Cu‐ and Zn‐loaded full‐length SOD1 isolated from erythrocytes (4.18 U/ng; Andersen et al. 1998; Marklund et al. 1997). Consequently, the N‐truncated SOD1 in CSF must be fully active to maintain this activity level. This observation suggests a native‐like structure and implies that proteolytic cleavage occurs in natively folded dimers (Bruns and Kopito 2007). Also, all SOD1 in CSF carries the native Cys57 to Cys146 intrasubunit disulfide bond (Figure 1d) which is important for structural integrity and enzymatic activity. Still, minute structural differences were observed. SOD1 with truncated subunits was separated from native forms by anion‐exchange chromatography implying subtle changes on the surface of the protein that effect the electrostatic forces and changes the interaction with the resin. We capitalized from this to separate the truncated subunits enough to do N‐terminal sequencing. We identified the truncation site to be after amino acid 26 by both sequencing and mass spectrometry. We successfully identified the cleaved 26‐residue N‐terminal peptide by mass spectrometry. Since immunocapture of native SOD1 also captured the short peptide, we could conclude that both parts are connected in the same structure. The ALS‐causing common A4V *SOD1* mutation illustrates the importance of structural integrity in the N‐terminus of the SOD1 protein. This mutation destabilizes both metalated and apo forms of the SOD1 protein and induces local unfolding in the β‐barrel, which promotes aggregation (Shaw et al. 2006). The small N‐terminal peptide must remain tightly associated within the native structure to avoid inducing comparable structural alterations.

We were able to demonstrate that the cleaved peptide stays connected to the SOD1 holoprotein and continues to stabilize its conformation and hence functionality. The N‐terminal peptide forms beta‐strand one and two in folded SOD1 and together with beta‐strand three and four forms the folding nucleus of SOD1 monomers (Nordlund and Oliveberg 2006) and is therefore the segment of SOD1 that should unfold last in the unfolding trajectory. Since the peptide normally is tightly bound to the remaining subunit (Figure 2c) it is not surprising that it can stay connected after cleavage as long as the dimer is intact. The present results do not indicate a correlation between the N‐truncated SOD1 molecule and misfolding (Figure 9). Following, these findings do not support a pathogenic role for the N‐terminal truncation in ALS or its involvement in the formation of the cytosolic SOD1 inclusions observed in ALS patients. This was confirmed by the finding that the truncation event is independent of ALS, since it was also detected in samples obtained from control individuals. We identified the N‐terminal side of Glycine 27 in the amino acid sequence as the truncation site and hence found out that the cleaved peptide contains the 26 most N‐terminal amino acids in the native SOD1 protein. The calculated molecular weight of the cleaved peptide is 2.6 kDa, which fits to the observation of the N‐truncation band ~3 kDa below the molecular weight of monomeric native SOD1 at 18 kDa.

Whether N‐truncated SOD1—either as individual fragments or incorporated into heterodimeric or homodimeric forms—possesses biological functions beyond its dismutase activity remains unknown, although it is conceivable. Given that the observed biochemical alterations in N‐truncated SOD1 are very subtle and that the levels are minute except in the CSF that is renewed multiple times per day, the truncation may not have significant biological implications. The fact that truncated SOD1 stays folded without release of the fragments could indicate that there is no biological function connected to the individual SOD1 fragments. Supporting this is the fact that we could not detect a comparable truncation in murine SOD1 (Figure 1b). If one or both fragments would have an important cellular function it would likely be conserved also in mice. However, the truncation process is not human‐specific since transgenic human SOD1 was found truncated in murine CSF although in small amounts (Figure 1c). This suggests that the truncation is sequence‐dependent and not conserved across species. We have not been able to analyze CSF from species with a conserved cleavage site (guinea pigs and nonhuman primates).

The fact that heterodimeric or homodimeric forms containing N‐truncated SOD1 excists may confer partial protection against unfolding, in contrast to homodimers of N‐truncated SOD1. Since we did not observe an increase in misfolding even in samples with a high proportion of N‐truncated SOD1, this could be attributable to the stabilizing effect of heterodimer formation.

How the N‐terminally cleaved SOD1 variant is formed is not clear. The PeptideCutter tool at the Expasy Bioinformatics Resource Portal (Gasteiger et al. 2005) could not identify any protease that would cut human SOD1 at the identified position between amino acids N26 and G27. However, hydroxylamine (NH_2_OH) chemically cleaves peptides at this exact sequence (Bornstein and Balian 1977) and N26/G27 is the only site in human SOD1. Hydroxylamine can cleave bovine SOD1 albeit at another position (Steinman et al. 1974). The in vitro condition for hydroxylamine cleavage is harsh (2 M NH2OH, pH 9, 45°C, 6 M guanidine as solvent, 4 h reaction time) and the likelihood of such a cleavage in vivo is negligible. Hence, the proteolytic mechanism responsible for the truncation remains unidentified.

SOD1 is part of a protein family characterized by conserved yet intrinsically disordered regions, which allows the protein to adapt different conformations in these regions and by doing so to execute different functions. Some ALS‐associated SOD1 mutations can interfere with this by reducing the degree of unsorted structures including the N‐terminal A4V mutation. How this alteration is connected to neurodegeneration has not been elucidated (Eleutherio et al. 2021). It is remarkable that SOD1 can carry a 26 amino acid truncation in the N‐terminus when C‐terminal truncations have a dramatic effect on stability and cause highly penetrant and early onset ALS (Jonsson et al. 2004). Our data do not indicate that N‐terminal truncation promotes SOD1 misfolding although a fraction of SOD1 in CSF does misfold at 42°C (Zetterström et al. 2011). Posttranslational modifications like glutathionylation (Marklund et al. 1997; Redler et al. 2011) could partly explain the misfolding.

## 5 Conclusion

This study identifies the N‐terminal truncation of SOD1 in CSF. Only trace amounts of truncated SOD1 were detected in other human tissues. Our findings suggest that N‐terminal truncation does not contribute to ALS pathogenesis. Notably, truncated SOD1 retains enzymatic activity, and the cleaved peptide remains associated within the native dimer structure. These observations indicate that the N‐terminal peptide contributes to structural stabilization of the SOD1 protein.

## Author Contributions

**Laura Leykam:** investigation, visualization, writing – original draft, writing – review and editing. **Karin M. E. Forsberg:** resources, writing – review and editing. **Peter M. Andersen:** resources, writing – review and editing, funding acquisition. **Thomas Brännström:** resources, writing – review and editing, funding acquisition. **Sophia Weiner:** investigation. **John Rönnholm:** investigation. **Kaj Blennow:** resources, writing – review and editing, funding acquisition. **Henrik Zetterberg:** resources, writing – review and editing, funding acquisition. **Stefan L. Marklund:** conceptualization, methodology, supervision, formal analysis, writing – review and editing. **Johan Gobom:** investigation, validation, writing – review and editing. **Per Zetterström:** conceptualization, project administration, writing – review and editing, writing – original draft, visualization, funding acquisition, formal analysis, investigation.

## Funding

The ALS Research Center at Umeå University and Umeå University Hospital is supported by grants from the Swedish Brain Foundation (grants nr. 2012‐0262, 2012‐0305, 2013‐0279, 2016‐0303, 2020‐0353), the Swedish Research Council (grants nr 2012‐3167, 2017‐03100), the Knut and Alice Wallenberg Foundation (grants nr. 2012.0091, 2014.0305, 2020.0232), the Ulla‐Carin Lindquist Foundation, the Neuroförbundet Association, Umeå University Insamlingsstiftelsen (223‐2808‐12, 223‐1881‐13, 2.1.12‐1605‐14, 2.1.6‐452‐20), Västerbotten County Council, King Gustaf V:s and Queen Victoria's Freemason's Foundation, and Fort Knox Charity Foundation/Olsson och Olsson Foundation. H.Z. is a Wallenberg Scholar and a Distinguished Professor at the Swedish Research Council supported by grants from the Swedish Research Council (#2023‐00356, #2022‐01018 and #2019‐02397), the European Union's Horizon Europe research and innovation programme under grant agreement No 101053962, Swedish State Support for Clinical Research (#ALFGBG‐71320), the Alzheimer Drug Discovery Foundation (ADDF), USA (#201809‐2016862), the AD Strategic Fund and the Alzheimer's Association (#ADSF‐21‐831376‐C, #ADSF‐21‐831381‐C, #ADSF‐21‐831377‐C, and #ADSF‐24‐1284328‐C), the European Partnership on Metrology, cofinanced from the European Union's Horizon Europe Research and Innovation Programme and by the Participating States (NEuroBioStand, #22HLT07), the Bluefield Project, Cure Alzheimer's Fund, the Olav Thon Foundation, the Erling‐Persson Family Foundation, Familjen Rönströms Stiftelse, Stiftelsen för Gamla Tjänarinnor, Hjärnfonden, Sweden (#FO2022‐0270), the European Union's Horizon 2020 research and innovation programme under the Marie Skłodowska‐Curie grant agreement No 860197 (MIRIADE), the European Union Joint Programme—Neurodegenerative Disease Research (JPND2021‐00694), the National Institute for Health and Care Research University College London Hospitals Biomedical Research Centre, the UK Dementia Research Institute at UCL (UKDRI‐1003), and an anonymous donor. K.B. is supported by the Swedish Research Council (#2017‐00915 and #2022‐00732), the Swedish Alzheimer Foundation (#AF‐930351, #AF‐939721, #AF‐968270, and #AF‐994551), Hjärnfonden, Sweden (#ALZ2022‐0006 and #FO2024‐0048‐TK‐130), the Swedish state under the agreement between the Swedish government and the County Councils, the ALF‐agreement (#ALFGBG‐965240 and #ALFGBG‐1006418), the European Union Joint Program for Neurodegenerative Disorders (JPND2019‐466‐236), the Alzheimer's Association 2021 Zenith Award (ZEN‐21‐848495), the Alzheimer's Association 2022‐2025 Grant (SG‐23‐1038904 QC), La Fondation Recherche Alzheimer (FRA), Paris, France, the Kirsten and Freddy Johansen Foundation, Copenhagen, Denmark, Familjen Rönströms Stiftelse, Stockholm, Sweden, and an anonymous philanthropist and donor.

## Conflicts of Interest

L.L., T.B., S.W., J.R., J.G., S.L.M., and P.Z.: nothing to declare. K.M.E.F.: Clinical trial site investigator for Amylyx, Biogen, Ionis Pharmaceuticals, PTC Pharmaceuticals, Sanofi, and ITB‐Med. P.M.A.: Paid consultancies and serve/have served on advisory boards for Biogen, Roche, Arrowhead, Avrion, Regeneron, uniQure, Voyager and Orphazyme A/S; clinical trial site investigator for AB Science, AL‐S Pharma and Lilly, Amylyx, Alexion Pharmaceuticals, Biogen Idec, IBT‐Med, IONIS Pharmaceuticals, Orion Pharma, PTC Pharmaceuticals, Sanofi. Since 1993 Director of the ALS‐genetic laboratory at Umeå University Hospital that performs not‐for‐profit research genetic testing and free genetic testing including for SOD1. Since 2021, member of the ClinGen ALS Gene variant Curation Expert panel. External advisor to the European Medicine Agency. H.Z. has served at scientific advisory boards and/or as a consultant for Abbvie, Acumen, Alector, Alzinova, ALZpath, Amylyx, Annexon, Apellis, Artery Therapeutics, AZTherapies, Cognito Therapeutics, CogRx, Denali, Eisai, Enigma, LabCorp, Merck Sharp & Dohme, Merry Life, Nervgen, Novo Nordisk, Optoceutics, Passage Bio, Pinteon Therapeutics, Prothena, Quanterix, Red Abbey Labs, reMYND, Roche, Samumed, ScandiBio Therapeutics AB, Siemens Healthineers, Triplet Therapeutics, and Wave, has given lectures sponsored by Alzecure, BioArctic, Biogen, Cellectricon, Fujirebio, LabCorp, Lilly, Novo Nordisk, Oy Medix Biochemica AB, Roche, and WebMD, is a cofounder of Brain Biomarker Solutions in Gothenburg AB (BBS), which is a part of the GU Ventures Incubator Program, and is a shareholder of CERimmune Therapeutics (outside submitted work). K.B. has served as a consultant and at advisory boards for Abbvie, AC Immune, ALZPath, AriBio, Beckman‐Coulter, BioArctic, Biogen, Eisai, Lilly, Moleac Pte. Ltd., Neurimmune, Novartis, Ono Pharma, Prothena, Quanterix, Roche Diagnostics, Sanofi and Siemens Healthineers; has served at data monitoring committees for Julius Clinical and Novartis; has given lectures, produced educational materials and participated in educational programs for AC Immune, Biogen, Celdara Medical, Eisai and Roche Diagnostics; and is a cofounder of Brain Biomarker Solutions in Gothenburg AB (BBS), which is a part of the GU Ventures Incubator Program, outside the work presented in this paper.

## Supporting information


**Data S1:** jnc70382‐sup‐0001‐Supinfo1.pdf.

## Data Availability

The data that support the findings of this study are available from the corresponding author upon reasonable request, if of scientific interest and legally and ethically possible.
