# Supplementary material for: N‐Truncated Superoxide Dismutase‐1 in Cerebrospinal Fluid Is Folded and Active
Source: J Neurochem. 2026 Feb 10;170(2):e70382. doi: 10.1111/jnc.70382 (PMC12887930; doi:10.1111/jnc.70382)
Supplement: Supplementary file 1 — Data S1: jnc70382‐sup‐0001‐Supinfo1.pdf. [file JNC-170-0-s001.pdf]

Supporting Information for:

N-truncated superoxide dismutase-1 in cerebrospinal fluid is folded and active

Laura Leykam [1], Karin M.E. Forsberg[2], Peter M. Andersen[2], Thomas Brännström[1], Sophia Weiner[3,4], John Rönnholm[3,4], Kaj Blennow[3,4,5,6], Henrik Zetterberg[3,4,7,8,9,10,11,12], Stefan L. Marklund[1], Johan Gobom[3,4], Per Zetterström[1*]

[1] Department of Medical Biosciences, Clinical Chemistry, Umeå University, S-901 85, Umeå, Sweden.
[2] Department of Clinical Sciences, Neurosciences, Umeå University, S-901 85 Umeå, Sweden.
[3]Institute of Neuroscience and Physiology, Department of Psychiatry and Neurochemistry, University of Gothenburg, Mölndal, Sweden.
[4]Clinical Neurochemistry Lab, Institute of Neuroscience and Physiology, Sahlgrenska University Hospital, Mölndal, Sweden.
[5]Paris Brain Institute, ICM, Pitié-Salpêtrière Hospital, Sorbonne University, Paris, France
[6]Neurodegenerative Disorder Research Center, Division of Life Sciences and Medicine, and Department of Neurology, Institute on Aging and Brain Disorders, University of Science and Technology of China and First Affiliated Hospital of USTC, Hefei, P.R. China
[7]Department of Neurodegenerative Disease, UCL Institute of Neurology, London, UK.
[8]UK Dementia Research Institute, UCL, London, UK.
[9]Hong Kong Center for Neurodegenerative Diseases, InnoHK, Hong Kong, China
[10]Wisconsin Alzheimer's Disease Research Center, University of Wisconsin School of Medicine and Public Health, University of Wisconsin-Madison, Madison, WI, USA.
[11]Department of Pathology and Laboratory Medicine, University of Wisconsin School of Medicine and Public Health, Madison, WI, USA.
[12]Centre for Brain Research, Indian Institute of Science, Bangalore, India.

* To whom correspondence should be addressed:

Per Zetterström

Department of Medical Biosciences, Clinical Chemistry

Umeå University

SE-901 85 Umeå, SWEDEN

Tel: +46 90 785 2950

Per.Zetterstrom@umu.se

Figure S1: Uncropped western blots

Figure 1a

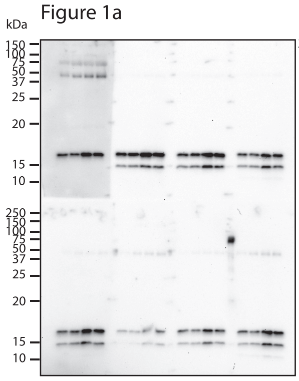

Figure 1b

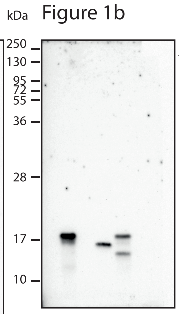

Figure 1c

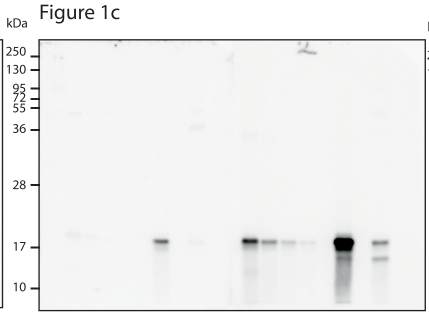

Figure 1d

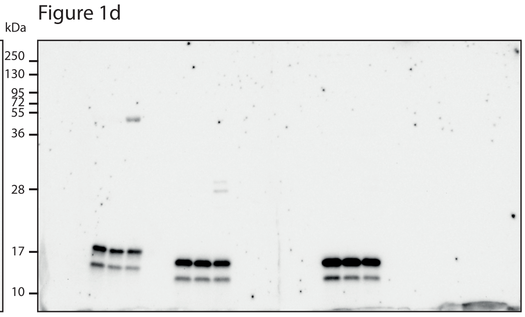

Figure 3a

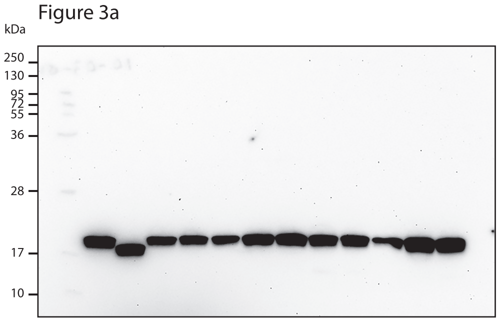

Figure 3b

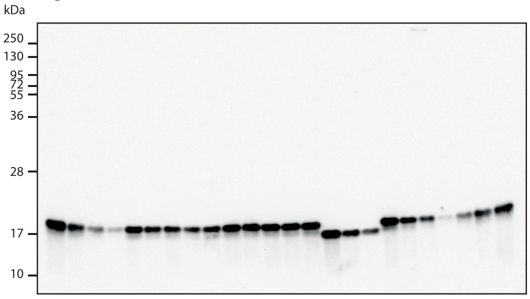

Figure 3c

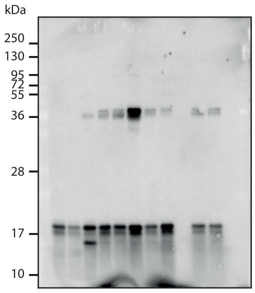

Figure 3d

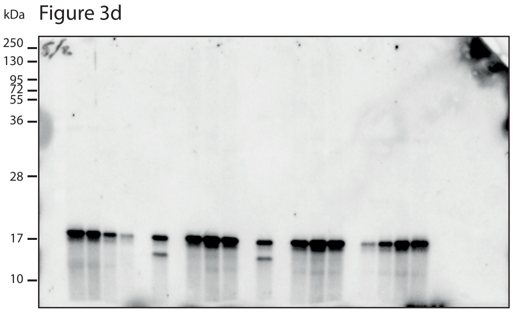

Figure 4

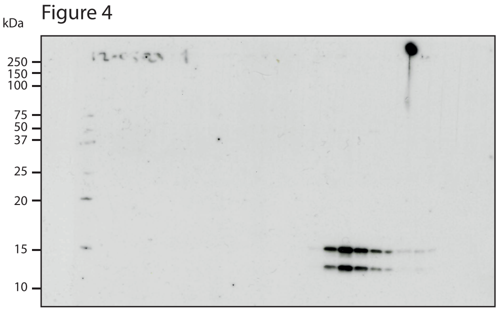

Figure 5

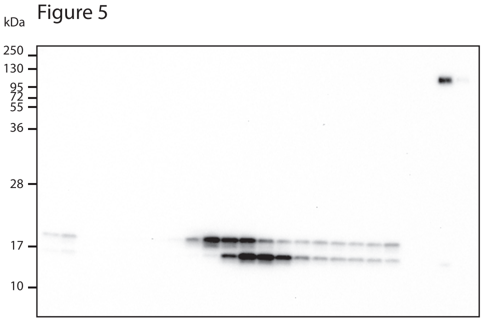

Figure 6

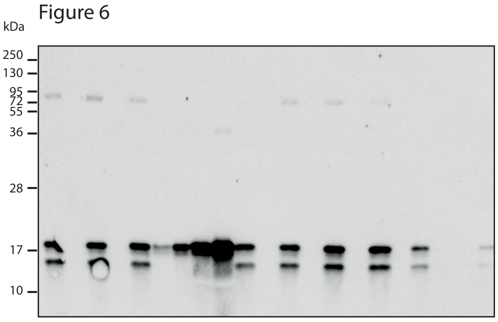

Figure 8

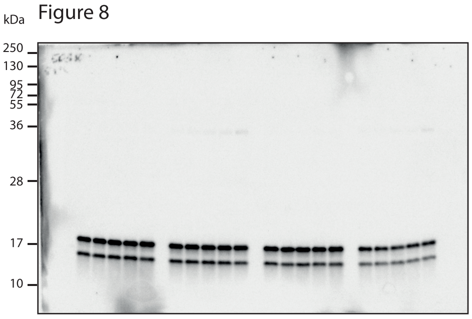

Figure 9

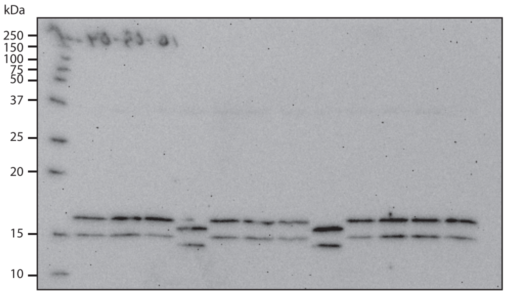

Supporting Table S1: Full statistical report

1. Establishing that selected ALS patients and controls with high and low levels of misfolded SOD1 are different.

Case Processing Summary

	ALS	Cases					
		Valid		Missing		Total	
		N	Percent	N	Percent	N	Percent
Agaz_misELISA	Control	6	100.0%	0	0.0%	6	100.0%
	ALS	6	100.0%	0	0.0%	6	100.0%
Alma_misELISA	Control	6	100.0%	0	0.0%	6	100.0%
	ALS	6	100.0%	0	0.0%	6	100.0%
Via_misELISA	Control	6	100.0%	0	0.0%	6	100.0%
	ALS	6	100.0%	0	0.0%	6	100.0%
CuZnSOD	Control	6	100.0%	0	0.0%	6	100.0%
	ALS	6	100.0%	0	0.0%	6	100.0%
Protein	Control	6	100.0%	0	0.0%	6	100.0%
	ALS	6	100.0%	0	0.0%	6	100.0%
Sample_date	Control	6	100.0%	0	0.0%	6	100.0%
	ALS	6	100.0%	0	0.0%	6	100.0%

Descriptives

	ALS			Statistic	Std. Error
Agaz_misELISA	Control	Mean		.0456	.01377
		95% Confidence Interval for Mean	Lower Bound	.0102	
			Upper Bound	.0810	
		5% Trimmed Mean		.0441	
		Median		.0389	
		Variance		.001	
		Std. Deviation		.03372	
		Minimum		.02	
		Maximum		.10	
		Range		.08	
		Interquartile Range		.05	
		Skewness		.963	.845
		Kurtosis		.119	1.741
	ALS	Mean		.0397	.00973
		95% Confidence Interval for Mean	Lower Bound	.0147	
			Upper Bound	.0647	

		5% Trimmed Mean		.0396	
		Median		.0384	
		Variance		.001	
		Std. Deviation		.02384	
		Minimum		.01	
		Maximum		.07	
		Range		.05	
		Interquartile Range		.04	
		Skewness		.053	.845
		Kurtosis		-2.856	1.741
Alma_misELISA	Control	Mean		.0555	.02411
		95% Confidence Interval for Mean	Lower Bound	-.0065	
			Upper Bound	.1175	
		5% Trimmed Mean		.0513	
		Median		.0343	
		Variance		.003	
		Std. Deviation		.05905	
		Minimum		.02	
		Maximum		.17	
		Range		.16	
		Interquartile Range		.07	
		Skewness		2.024	.845
		Kurtosis		4.297	1.741
	ALS	Mean		.0468	.01044
		95% Confidence Interval for Mean	Lower Bound	.0199	
			Upper Bound	.0736	
		5% Trimmed Mean		.0470	
		Median		.0489	
		Variance		.001	
		Std. Deviation		.02556	
		Minimum		.02	
		Maximum		.07	
		Range		.06	
		Interquartile Range		.05	
		Skewness		-.111	.845
		Kurtosis		-2.841	1.741
Via_misELISA	Control	Mean		.0465	.01374
		95% Confidence Interval for Mean	Lower Bound	.0112	
			Upper Bound	.0818	
		5% Trimmed Mean		.0454	
		Median		.0374	

		Variance		.001	
		Std. Deviation		.03365	
		Minimum		.01	
		Maximum		.10	
		Range		.09	
		Interquartile Range		.06	
		Skewness		.738	.845
		Kurtosis		-.941	1.741
	ALS	Mean		.0364	.00721
		95% Confidence Interval for Mean	Lower Bound	.0178	
			Upper Bound	.0549	
		5% Trimmed Mean		.0365	
		Median		.0402	
		Variance		.000	
		Std. Deviation		.01767	
		Minimum		.01	
		Maximum		.06	
		Range		.04	
		Interquartile Range		.04	
		Skewness		-.332	.845
		Kurtosis		-2.233	1.741
CuZnSOD	Control	Mean		603.3333	223.18448
		95% Confidence Interval for Mean	Lower Bound	29.6194	
			Upper Bound	1177.0473	
		5% Trimmed Mean		592.7963	
		Median		522.1667	
		Variance		298867.867	
		Std. Deviation		546.68809	
		Minimum		106.33	
		Maximum		1290.00	
		Range		1183.67	
		Interquartile Range		1016.42	
		Skewness		.198	.845
		Kurtosis		-2.743	1.741
	ALS	Mean		407.5528	202.37475
		95% Confidence Interval for Mean	Lower Bound	-112.6681	
			Upper Bound	927.7736	
		5% Trimmed Mean		387.6883	
		Median		104.1583	
		Variance		245733.231	
		Std. Deviation		495.71487	

		Minimum		62.67	
		Maximum		1110.00	
		Range		1047.33	
		Interquartile Range		933.58	
		Skewness		.994	.845
		Kurtosis		-1.696	1.741
Protein	Control	Mean		341.9621	33.02179
		95% Confidence Interval for Mean	Lower Bound	257.0769	
			Upper Bound	426.8473	
		5% Trimmed Mean		347.4901	
		Median		363.9534	
		Variance		6542.631	
		Std. Deviation		80.88653	
		Minimum		181.80	
		Maximum		402.62	
		Range		220.83	
		Interquartile Range		84.23	
		Skewness		-2.126	.845
		Kurtosis		4.827	1.741
	ALS	Mean		357.8785	25.86076
		95% Confidence Interval for Mean	Lower Bound	291.4013	
			Upper Bound	424.3557	
		5% Trimmed Mean		358.1001	
		Median		358.4236	
		Variance		4012.672	
		Std. Deviation		63.34565	
		Minimum		267.49	
		Maximum		444.28	
		Range		176.78	
		Interquartile Range		111.20	
		Skewness		-.089	.845
		Kurtosis		-.574	1.741
Sample_date	Control	Mean		12-JAN-97	424 02:42:21,912
		95% Confidence Interval for Mean	Lower Bound	17-JAN-94	
			Upper Bound	07-JAN-00	
		5% Trimmed Mean		28-JAN-97	
		Median		30-DEC-97	
		Variance		805640703897600 0.000	
		Std. Deviation		1038 20:38:10,155	
		Minimum		10-JUN-93	

				Statistic	Std. Error
		Maximum		01-NOV-99	
		Range		2335 00:00:00	
		Interquartile Range		2125 18:00:00	
		Skewness		-.600	.845
		Kurtosis		-2.030	1.741
	ALS	Mean		09-JUL-02	398 06:12:42,779
		95% Confidence Interval for Mean	Lower Bound	20-SEP-99	
			Upper Bound	28-APR-05	
		5% Trimmed Mean		22-JUN-02	
		Median		02-JAN-02	
		Variance		710410806374400 0.000	
		Std. Deviation		975 12:44:31,080	
		Minimum		03-JUN-99	
		Maximum		27-JUN-06	
		Range		2581 00:00:00	
		Interquartile Range		1774 18:00:00	
		Skewness		.510	.845
		Kurtosis		-1.034	1.741

2. Explore data for levels of SOD1 variants including test of normality

Case Processing Summary

		Cases					
		Valid		Missing		Total	
	misELISA	N	Percent	N	Percent	N	Percent
SOD1upperband	low	6	100.0%	0	0.0%	6	100.0%
	high	6	100.0%	0	0.0%	6	100.0%
SOD1lowerband	low	6	100.0%	0	0.0%	6	100.0%
	high	6	100.0%	0	0.0%	6	100.0%

Descriptives

	misELISA			Statistic	Std. Error
SOD1upperband	low	Mean		1518.8606	124.01595
		95% Confidence Interval for Mean	Lower Bound	1200.0674	
			Upper Bound	1837.6537	
		5% Trimmed Mean		1511.9230	
		Median		1421.8443	
		Variance		92279.736	

		Std. Deviation		303.77580	
		Minimum		1197.28	
		Maximum		1965.32	
		Range		768.04	
		Interquartile Range		582.07	
		Skewness		.721	.845
		Kurtosis		-1.248	1.741
	high	Mean		1797.8680	277.13195
		95% Confidence Interval for Mean	Lower Bound	1085.4777	
			Upper Bound	2510.2584	
		5% Trimmed Mean		1775.2128	
		Median		1722.9605	
		Variance		460812.695	
		Std. Deviation		678.83186	
		Minimum		997.11	
		Maximum		3006.42	
		Range		2009.30	
		Interquartile Range		886.98	
		Skewness		1.141	.845
		Kurtosis		2.216	1.741
SOD1lowerband	low	Mean		797.5033	96.67167
		95% Confidence Interval for Mean	Lower Bound	549.0009	
			Upper Bound	1046.0058	
		5% Trimmed Mean		793.0032	
		Median		748.9559	
		Variance		56072.476	
		Std. Deviation		236.79628	
		Minimum		495.52	
		Maximum		1180.49	
		Range		684.97	
		Interquartile Range		362.50	
		Skewness		.643	.845
		Kurtosis		.533	1.741
	high	Mean		857.7234	135.96383
		95% Confidence Interval for Mean	Lower Bound	508.2172	
			Upper Bound	1207.2295	
		5% Trimmed Mean		854.4013	
		Median		844.6351	
		Variance		110916.984	
		Std. Deviation		333.04202	
		Minimum		455.89	

	Maximum	1319.35	
	Range	863.47	
	Interquartile Range	664.19	
	Skewness	.199	.845
	Kurtosis	-1.257	1.741

Tests of Normality

		Kolmogorov-Smirnov[a]			Shapiro-Wilk		
	misELISA	Statistic	df	Sig.	Statistic	df	Sig.
SOD1upperband	low	.250	6	.200*	.904	6	.395
	high	.259	6	.200*	.920	6	.507
SOD1lowerband	low	.163	6	.200*	.968	6	.878
	high	.165	6	.200*	.949	6	.735

*. This is a lower bound of the true significance.

a. Lilliefors Significance Correction

3. Independent t-test for levels of SOD1 variants

Group Statistics

	misELISA	N	Mean	Std. Deviation	Std. Error Mean
SOD1upperband	low	6	1518.8606	303.77580	124.01595
	high	6	1797.8680	678.83186	277.13195
SOD1lowerband	low	6	797.5033	236.79628	96.67167
	high	6	857.7234	333.04202	135.96383

Independent Samples Test

		Levene's Test for Equality of Variances		t-test for Equality of Means							
						Significance		Mean Difference	Std. Error Difference	95% Confidence Interval of the Difference	
		F	Sig.	t	df	One-Sided p	Two-Sided p			Lower	Upper
SOD1upperband	Equal variances assumed	1.180	.303	-.919	10	.190	.380	-279.00745	303.61501	-955.50384	397.48894
	Equal variances not assumed			-.919	6.925	.195	.389	-279.00745	303.61501	-998.51497	440.50008
SOD1lowerband	Equal variances assumed	.593	.459	-.361	10	.363	.726	-60.22003	166.82799	-431.93595	311.49589
	Equal variances not assumed			-.361	9.026	.363	.726	-60.22003	166.82799	-437.44325	317.00319

Independent Samples Effect Sizes

		Standardizer[a]	Point Estimate	95% Confidence Interval	
				Lower	Upper
SOD1upperband	Cohen's d	525.87662	-.531	-1.673	.637
	Hedges' correction	569.90422	-.490	-1.543	.587
	Glass's delta	678.83186	-.411	-1.550	.766
SOD1lowerband	Cohen's d	288.95455	-.208	-1.338	.932
	Hedges' correction	313.14649	-.192	-1.235	.860
	Glass's delta	333.04202	-.181	-1.309	.965

a. The denominator used in estimating the effect sizes.

Cohen's d uses the pooled standard deviation.

Hedges' correction uses the pooled standard deviation, plus a correction factor.

Glass's delta uses the sample standard deviation of the control (i.e., the second) group.
